# Reactions of aryl dimethylphosphinothioate esters with anionic oxygen nucleophiles: transition state structure in 70% water–30% ethanol†

**DOI:** 10.1039/d0ra10759j

**Published:** 2021-02-25

**Authors:** Georgina I. Kalu, Collins I. Ubochi, Ikenna Onyido

**Affiliations:** Department of Chemistry, Imo State University Owerri Nigeria; Department of Pure and Industrial Chemistry, Nnamdi Azikiwe University Awka Nigeria ikennaonyido@yahoo.com +234-806-268-5122

## Abstract

Aryl dimethylphosphinates, 2, react with anionic oxygen nucleophiles in water *via* a concerted (A_N_D_N_) mechanism. With EtO^−^ in anhydrous ethanol, the mechanism is associative (A_N_ + D_N_), with rate-limiting pentacoordinate intermediate formation. This change in mechanism with solvent change has been ascribed to changes in the nucleophile and leaving group basicities accompanying solvent change. This paper reports on a kinetic analysis of the reactions of the aryl dimethylphosphinothioates, 3a–g, with oxygen nucleophiles in 70% water–30% ethanol (v/v) solvent at 25 °C, reactions known to proceed by a concerted mechanism in water, to test the rationalization stated above, since the nucleophiles and LGs of interest are more basic in aqueous ethanol than in water. The change in solvent causes an *ca.* 14 to 320-fold decrease in rate. Hammett and Brønsted-type correlations characterize a concerted TS with less P–LG bonding in aqueous ethanol than in water. Two opposing consequences are associated with the solvent change: (a) increased basicity of nucleophiles and LGs, which lead to a modest tightening of the TS; and (b) better stabilization of the IS relative to the TS in aqueous ethanol, which results in a slower reaction with a more product-like TS. Hammond and anti-Hammond effects on the TS arising from better stabilization of the IS over the TS dominate over the effects of increased nucleophile and LG basicity in determining the looser TS structure in aqueous ethanol. An altered TS structure is consistent with an altered reaction potential energy surface, in this case caused by a change in solvent polarity.

## Abstract 2

Solvent stabilization of initial state (along *x*, *y* axis) leads to looser TS (vector *z*).

## Graphical abstract

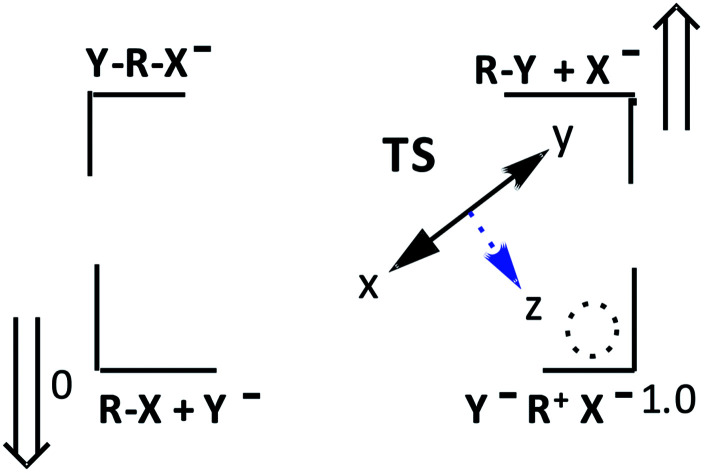

## Introduction

Our interest in the transfer of the (thio)phosphinoyl group^1^ between oxyanionic nucleophiles in hydroxylic solvents^2–5^ is based, in part, on the basic structural relationship of these substrates with (thio)phosphonate and (thio)phosphate esters frequently encountered in uncatalyzed and biological phosphoryl transfers.^6^ Even though phosphinate esters do not occur naturally, some phosphinic acids have found application as inhibitors of metalloproteases^7^ and as medicinal agents towards some diseases.^8^ The elucidation of the structure of the transition states of the reactions of phosphinate esters and other organophosphorus esters with a variety of nucleophiles in solution chemistry has contributed to the present status of understanding of the mechanistic details of phosphoryl transfer reactions.^5,9–12^ Of particular interest are the questions of whether biological phosphoryl transfers are concerted or stepwise mechanisms involving intermediates, and whether the TS in a chemical reaction is altered by a mediating enzyme in the analogous biochemical processes.^13^ It is also of interest to understand the factors that predispose reacting systems to the choices they make regarding their operational mechanisms and rate-limiting transition states under various reaction conditions.^14,15^

**Scheme 1:**
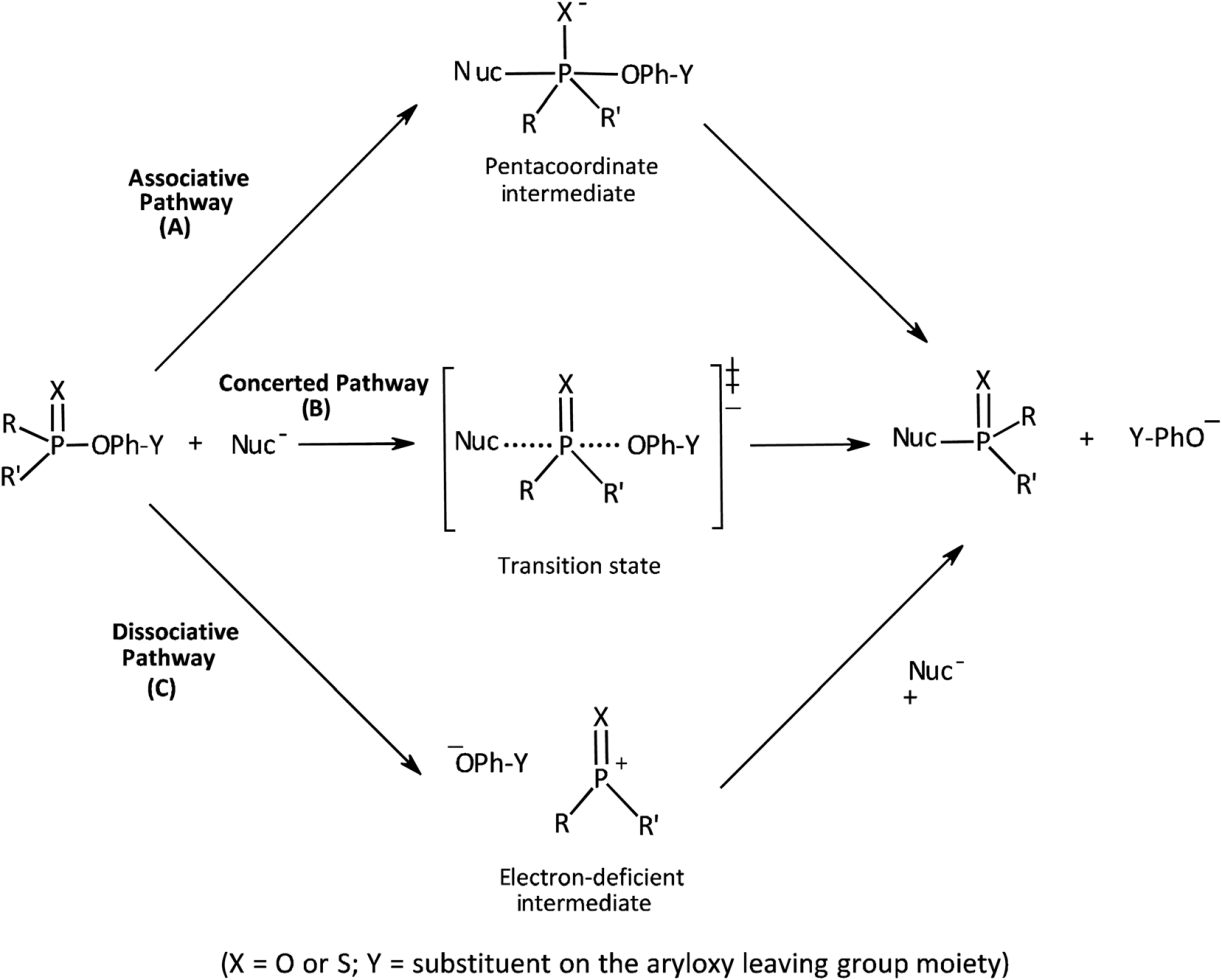


**Fig. 1:**
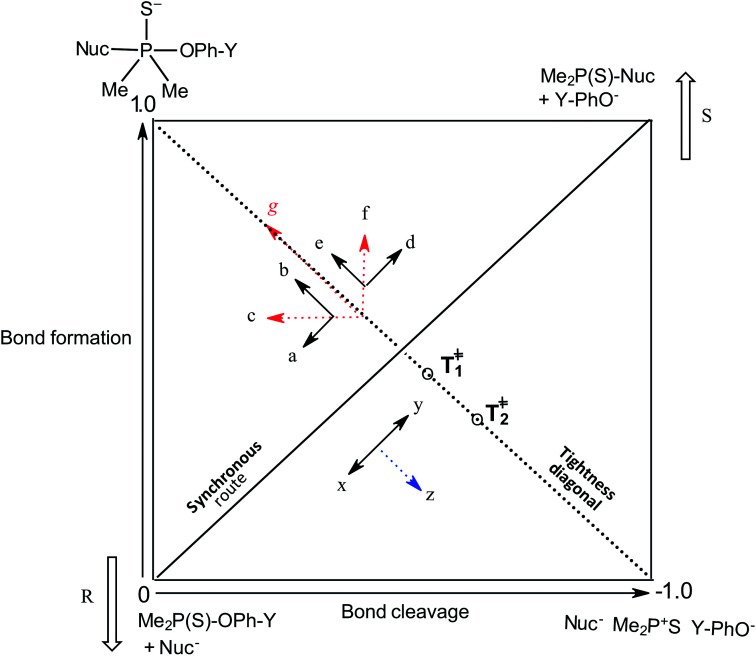
More O'Ferrall–Jencks diagram for the transfer of the thiophosphinoyl group between the nucleophile and an aryloxide LG. Extent of bond formation and bond fission is measured along the vertical and horizontal axes, respectively (see text). The TS in water, *T*^‡^_1_, is slightly displaced from the intersection of the synchronous route and tightness diagonal (see ref. 2). Change of solvent to ethanol moves the TS (resultant vector *g*) towards the associative corner (see text and ref. 4). Solvent change from water to 70% water–30% ethanol will slide *T*^‡^_1_ in the same direction but to a diminished extent due to basicity changes in nucleophile and LG. The reactant corner is stabilized by the aqueous ethanol solvent; this perturbation (lowering the reactant corner – bold arrow R – and raising the product corner – bold arrow S) would slide the TS along the reaction coordinate (*x* ↔ *y* motion) as the Hammond effect. The resultant movement perpendicular to the reaction coordinate, *i.e.* vector *z* = the anti-Hammond effect, would move the TS towards a looser TS, *T*^‡^_2_ (this work).

The three possible mechanisms^9^ for the transfer of a phosphinoyl or thiophosphinoyl group^1^ between two nucleophiles are given in Scheme 1 in which X could be O or S, while Y represents one or more substituents on the aryloxy leaving group (LG) moiety. Pathways A, B and C in this scheme describe a stepwise associative (A_N_ + D_N_) mechanism involving a pentacoordinate phosphorane-type intermediate whose formation or decomposition could be the rate-limiting step, a concerted (A_N_D_N_) mechanism involving the rate-limiting synchronous formation and breaking of bonds, and a stepwise dissociative (D_N_ + A_N_) mechanism typically involving the rate-limiting ionization of the substrate to yield a metaphosphate-type intermediate, respectively. These mechanisms are also shown in the More O'Ferrall–Jencks diagram^15,16^ in Fig. 1 in which the intermediates formed in the associative and dissociative pathways are located at the top left corner and lower right corner of the diagram, respectively, while the diagonal from the lower left (reactant) corner to the upper right (product) corner defines the concerted pathway.

Rate data for the reactions of anionic oxygen nucleophiles (HO^−^, RO^−^, Y-PhO^−^) with aryl diphenylphosphinates (Ph_2_P(=O)–OPh-Y, 1) and aryl dimethylphosphinates (Me_2_P(=O)–OPh-Y, 2) demonstrate an interesting mechanistic dichotomy in water and ethanol solvents. The alkaline hydrolysis of 1 in pure water^17,18^ and in a variety of water-containing binary solvent systems,^17–21^ as well as the ethanolysis of the same substrate series in pure ethanol,^22^ proceeds *via* the associative pathway, with rate-limiting formation of a pentacoordinate intermediate. The reaction of the same substrate series with the less basic phenoxide ion (PhO^−^) proceeds by a concerted mechanism in water.^23^ When the solvent is changed to ethanol, the mechanism for the reaction with PhO^−^ as nucleophile becomes associative, with rate-limiting formation of the pentacoordinate intermediate.^24^

The same mechanistic dichotomy outlined above for 1 is also encountered in the reactions of the Me_2_P(=O)–OPh-Y series of compounds, 2, with the anionic oxygen nucleophiles EtO^−^ and HO^−^. Whereas the reaction of 2 with EtO^−^ in pure ethanol occurs *via* a stepwise mechanism with rate-limiting formation of a pentacoordinate intermediate,^4^ its reaction with HO^−^ in 90% water–10% dioxane occurs by a concerted S_N_2(P)-type mechanism, with Brønsted *β*_nuc_ = 0.41.^25^ A value of *β*_lg_ = −0.47 was calculated^4^ from the original data^25^ for this reaction from which the Leffler indices^26^*α*_bf_ = *β*_nuc_/*β*_eq_ = 0.47 and *α*_br_ = *β*_lg_/*β*_eq_ = −0.53 were obtained for bond formation and bond breaking,^27^ respectively, according to eqn (1), with *β*_eq_ defined in eqn (2). These Leffler parameters locate the TS for the reactions of 2 with anionic oxygen nucleophiles in aqueous dioxane at *T*^‡^_1_ along the tightness diagonal, but slightly displaced from the intersection between the synchronous route and the tightness diagonal in Fig. 1.1
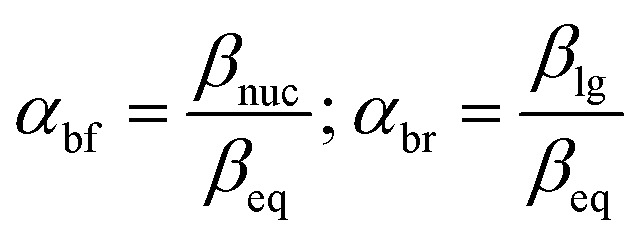

2 *β*_nuc_ − *β*_lg_ = *β*_eq_

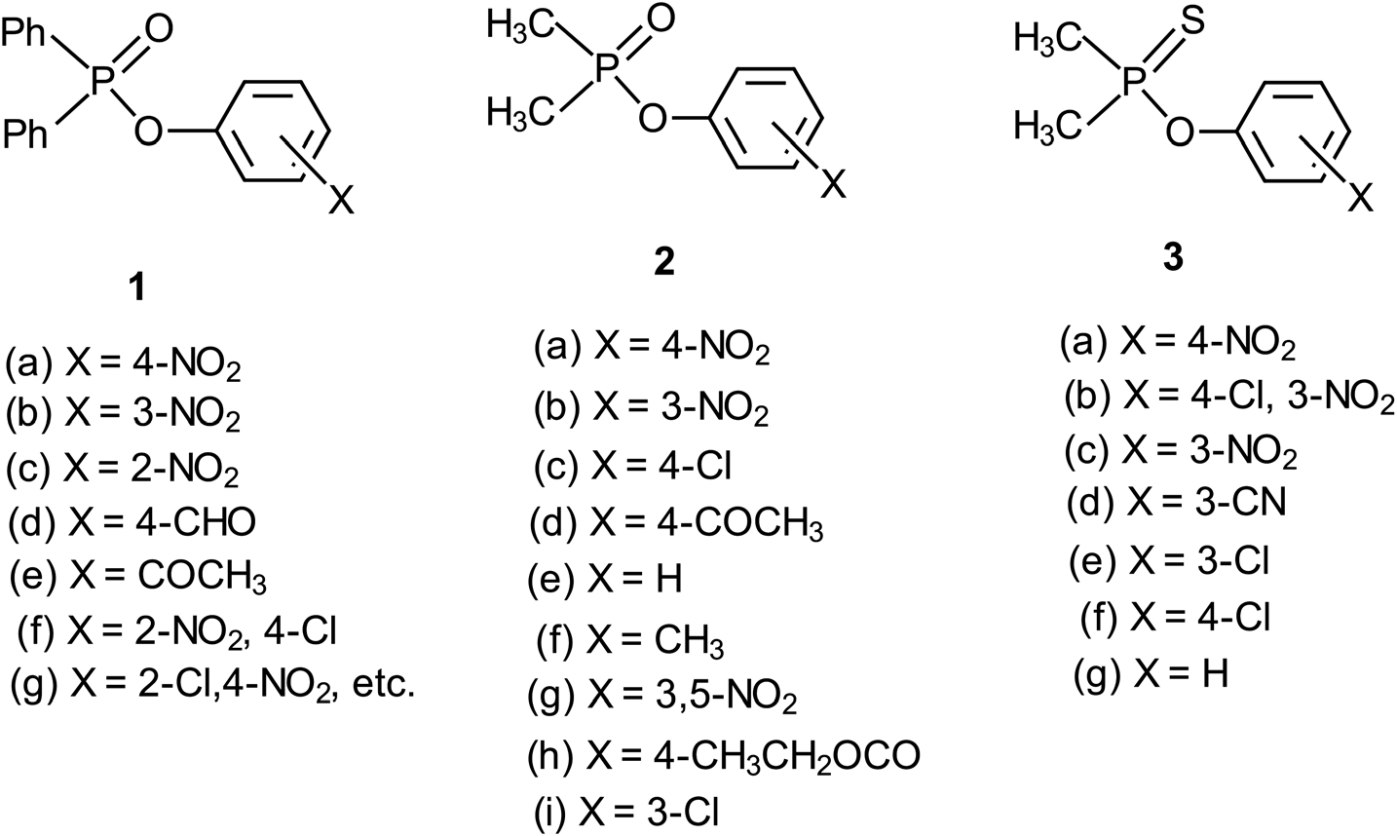


The p*K*_a_ values of the nucleophiles HO^−^, RO^−^, PhO^−^ and other substituted phenoxides in water and ethanol are listed in Table 1 from which it is seen that the nucleophiles and leaving groups utilized in the studies cited above are more basic in ethanol than in water. It has been explained^4^ that in this reaction system, the change in nucleophile and LG basicity, Δp*K*_a_ ≥ 3 and ≥5, respectively, caused by changing the solvent from water to ethanol, induces the observed change of mechanism in ethanol, by moving the concerted TS toward more associative TS structures with greater bond formation and little or no bond cleavage. This explanation is amplified below using the More O'Ferrall–Jencks diagram in Fig. 1, in order to establish the rationale for the present study.

**Table 1:** p*K*_a_ values of anionic nucleophiles in water, ethanol and 70% water–30% ethanol

Nucleophile	p*K*_a_ (H_2_O)^a^	p*K*_a_ (H_2_O–EtOH 70 : 30)^b^^,^^c^	p*K*_a_ (EtOH)
EtO^−^	16.0	16.81	19.18^d^
HO^−^	15.74	16.60	
CHCl_2_CH_2_O^−^	12.89	13.53 (13.52)^e^	
CF_3_CH_2_O^−^	12.43	12.96 (12.96)^e^	
CF_3_CF_2_CF_2_CH_2_O^−^	11.40	12.06	
4-MeOPhO^−^	10.20	10.83	
PhO^−^	9.95	10.54 (10.57)^e^	15.76^d^
4-ClPhO^−^	9.38	10.02 (10.03)^e^	14.90^d^ (14.80)^f^
3-ClPhO^−^	9.02	9.58	
3-CNPhO^−^	8.61	8.92 (8.89)^e^	
4-CNPhO^−^	7.95	8.30 (8.29)^e^	13.04^d^
2,5-Cl_2_PhO^−^	7.51	7.82	
2,4,5-Cl_3_PhO^−^	6.72	7.04	
2,3,5,6-F_4_PhO^−^	5.53	5.86 (5.86)^e^	

a Values at 25 °C, taken from W. P. Jencks and J. Regenstein, in *Handbook of Biochemistry*, ed. H. A. Sober, The Chemical Rubber Co., Cleveland, 1970, 2nd edn, section J-187.

b The aqueous ethanol solvent is 70% water–30% ethanol.

c These p*K*_a_ values were obtained as described in the Experimental section.

d Values at 25 °C, taken from I.-H. Um, Y.-J. Hong and D.-S. Kwon, *Tetrahedron*, 1997, **53**, 5073.

e Values in 70% water–30% ethanol at 25 °C measured by A. C. Hengge and R. Hoff, personal communication of to be published results.

f Value at 22 °C, given by G. Guanti, G. Cevasco, S. Thea, C. Dell'Erba and G. Petrillo, *J. Chem. Soc., Perkin Trans. 2*, 1981, 327.

For the reaction of 2 with oxyanionic nucleophiles, the change in the basicity of the nucleophiles in going from water to ethanol is thought^4^ to lower the bottom corners in Fig. 1. This perturbation slides the concerted TS *T*^‡^_1_ parallel to the reaction coordinate toward the bottom left corner (arrow a), the so-called Hammond effect. The same perturbation would also slide *T*^‡^_1_ in a direction perpendicular to the reaction coordinate, toward the left top associative corner (arrow b), *i.e.* the anti-Hammond effect. The resultant vector (arrow *c*) makes *β*_lg_ less negative, which is equivalent to a diminished separation of the leaving group in the TS in ethanol. The same argument has been advanced for the effect of solvent change, from water to ethanol, on the basicity of the aryloxide leaving groups in the substrate. Aryloxide ions are poorer leaving groups in ethanol, thus raising the energy of the right side of Fig. 1, compared to the left, a perturbation that moves *T*^‡^_1_ to the upper right as the Hammond effect (arrow d), while the anti-Hammond effect moves *T*^‡^_1_ to the upper left (arrow e), to produce the arrow *f* as the resultant vector. This movement results in a higher value of *β*_nuc_, *i.e.* greater bond formation between the nucleophile and the reaction site, with increased basicity of the leaving group. These responses of *β*_lg_ and *β*_nuc_ to enhanced basicity of the nucleophile and LG, respectively, which move the TS in the direction of the associative corner in Fig. 1 (with vector *g* as the resultant of vectors *c* and *f*), are a consequence of the positive cross interaction between the nucleophile and the LG in a concerted TS, quantified^28^ as the cross-interaction coefficient, *p*_*xy*_, defined in eqn (3).3
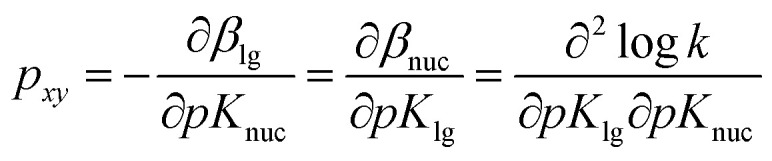


The above rationalization of the change in mechanism observed with solvent change from water to ethanol in these systems does not consider solvent polarity as an important variable that could affect reaction mechanisms and TS structures, even though the solvents of interest have significantly different polarities, as measured by their dielectric constants, *D* = 80.2 and 25.2 for water and ethanol, respectively, at 20 °C.^29,30^ Solvent and solvation effects are known to affect reactions in profound and, sometimes, complex ways.^31^ Bickelhaupt and coworkers^32^ have shown that the shapes of the potential energy surface of S_N_2-type processes are changed by solvation in ways that depend on solvent polarity and the way charges are distributed over the interacting entities, while the work of Carvalho *et al.*^33^ has highlighted the interplay between nucleophile charge and TS solvation in determining reactivities in the S_N_2(P) reactions of simple phosphoryl systems. In the reactions of phosphinate esters with anionic oxygen nucleophiles considered above, the polarities of water and ethanol in which the nucleophilic substitution reaction occurs through concerted and associative mechanisms, respectively, could be regarded as lying at two extremes. In the continuum of polarities between pure water and pure ethanol, the same reaction could conceivably occur with different transition states, such that a spectrum of transition states could occur in the progression from pure water, through water–ethanol mixtures of increasing ethanol content, to pure ethanol. The existence of a continuum of transition states in phosphoryl transfer reactions, in which bond formation and bond rupture are not necessarily synchronous, has been advanced.^9^

Three events are likely to occur in such a system as the ethanol content of the binary solvent increases from 0% in pure water to 100% in pure ethanol: (i) the strength of the nucleophile increases across the continuum of decreasing polarities due to increasing desolvation of the nucleophile; (ii) the strength of the P–LG bond becomes stronger as the LG becomes poorer (*i.e.* more basic) with decreasing polarity of the medium; and (iii) the reaction rate responds to decreasing polarity of the solvent, the specific response depending on the extent to which the initial state is stabilized or destabilized relative to the TS, with the consequential Hammond effects on the TS across the continuum of decreasing polarities. Such a study would be of interest because of the resulting information on the responses of the TS to changing solvent polarity and nucleophile/LG basicity, which could also shed some light on the factors that lead to changes in mechanism and/or TS structures in these reactions. These outcomes could be relevant in the discussion of mechanisms and transition states in uncatalyzed and biological phosphoryl transfer processes. Although studies of solvent effects on the reactions of phosphorus esters with a variety of nucleophiles have been reported^34,35^ the effects of solvent polarity on transition state structures of these reactions have not been specifically examined, to our knowledge.

We chose to study the effects of nucleophile/LG basicity and solvent polarity on the reactions of 3, thio analogues of 2, with the anionic oxygen nucleophiles HO^−^, alkoxides (RO^−^) and phenoxides (Y-PhO^−^) in 70% water–30% ethanol (v/v) with the specific objective of probing the TS structure using Hammett and Brønsted correlations. An earlier Brønsted and heavy atom kinetic isotope effect (KIE) study of the nucleophilic reaction of 3 with anionic oxygen nucleophiles in water showed^2^ unambiguously that the reaction proceeds *via* a concerted mechanism in which bond fission is slightly ahead of bond making in the TS (*β*_nuc_ = 0.47, *β*_lg_ = −0.53). Values of the Leffler indices *α*_bf_ = 0.47 and *α*_br_ = −0.53 obtained from the Brønsted parameters for this concerted reaction locate its TS at *T*^‡^_1_ in Fig. 1, exactly the same TS structure for the reaction of its oxygen analogue, 2.^4,25^ The similarity in the TS structure for the reactions of 2 and 3 in water is entirely fortuitous since *β*_nuc_, *β*_lg_ and *β*_eq_ values for their reactions in this solvent are significantly dissimilar.^4^ As can be seen in Table 1, the p*K*_a_ values of the nucleophiles and LGs employed in this and our earlier study^2^ are higher in 70% water–30% ethanol than in water. The results presented and discussed below with the aid of the More O'Ferrall–Jencks reaction map in Fig. 1 show that the concerted reaction TS is looser in the less polar solvent, in which case the change in solvent polarity has a dominant effect over solvent-induced changes in nucleophile and LG basicity in determining the TS structure in this solvent.

## Results and discussion

**Fig. 2:**
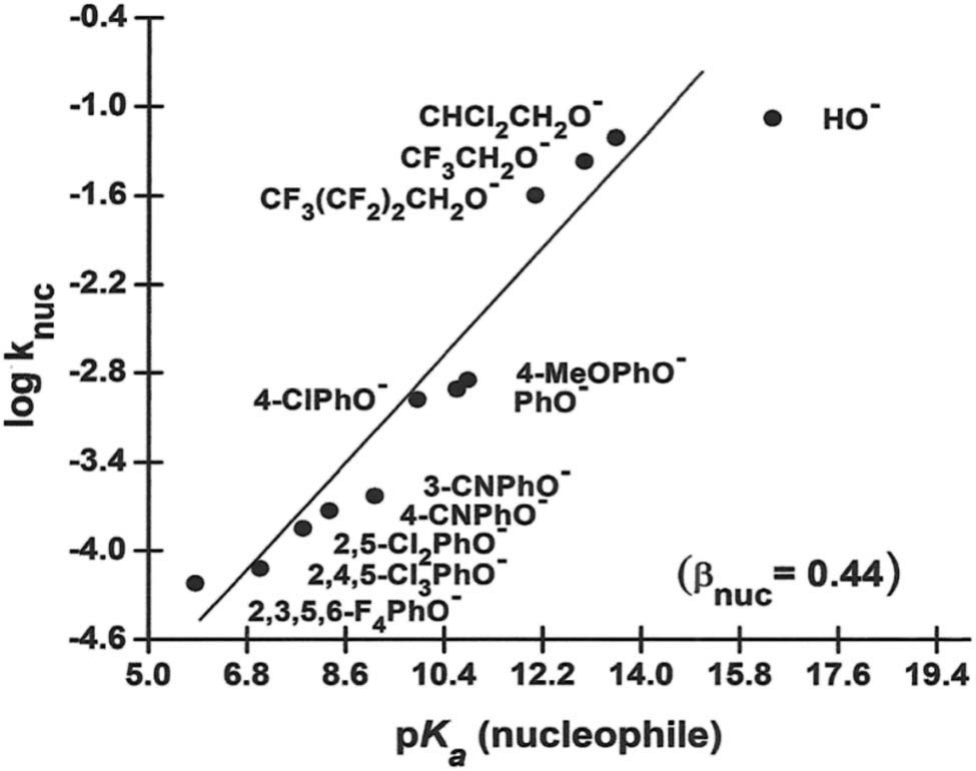
Plot of log *k*_nuc_*vs.* p*K*_a_ (nucleophile) for the reaction of 3a with oxyanionic nucleophiles in 70% water–30% ethanol (v/v) solvent at 25 °C. The line is defined by eqn (4). The point for HO^−^ shows a negative deviation and has been excluded in the calculation of *β*_nuc_ according to eqn (4) (see text).

The rates of the nucleophilic displacement reaction of oxyanions of the type HO^−^, RO^−^ and X-PhO^−^ (X = H and other substituents) with 4-nitrophenyl dimethylphosphinothioate ester, 3a, in 70% water–30% ethanol (v/v) were measured spectrophotometrically at 25 °C under pseudo-first-order conditions. The pseudo-first-order rate constants, *k*_obs_, calculated from linear plots of log(*A*_∞_ − *A*_*t*_) *versus* time, are given as ESI.† Second-order rate constants, *k*_nuc_, calculated from the linear plots of *k*_obs_*versus* nucleophile concentration for the different nucleophiles, are presented in Table 2, along with the p*K*_a_ values of the nucleophiles in this solvent. These rate constants were used to obtain the Brønsted-type plots of log *k*_nuc_*versus* p*K*_a_ (nucleophile) shown in Fig. 2.

**Table 2:** Second-order rate constants (*k*_nuc_)^a^ for the reaction of several oxygen nucleophiles with 4-nitrophenyl dimethylphosphinothioate (3a) in 70% water–30% ethanol (v/v) at 25 °C

Entry	Nucleophile	p*K*_a_^b^	10^4^*k*_nuc_^c^/M^−1^ s^−1^	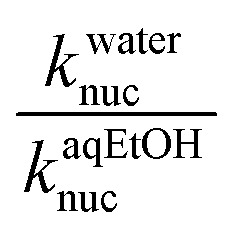 d^,^ e
1	HO^−^	16.60	830	79.9
2	CHCl_2_CH_2_O^−^	13.53	616	68.9
3	CF_3_CH_2_O^−^	12.96	427	89.2
4	CF_3_CF_2_CF_2_CH_2_O^−^	12.06	252	119.4
5	4-MeOPhO^−^	10.83	14.1	158.2
6	PhO^−^	10.54	12.3	43.9
7	4-ClPhO^−^	10.02	10.4	28.2
8	3-CNPhO^−^	8.92	2.37	86.1
9	4-CNPhO^−^	8.30	1.85	67.6
10	2,5-Cl_2_PhO^−^	7.82	1.43	69.9
11	2,4,5-Cl_3_PhO^−^	7.04	0.75	23.1
12	2,3,5,6-F_4_PhO^−^	5.86	0.60	13.7

a These rate constants were measured at ionic strength, *I* = 1.0 M (KCl).

b Values of p*K*_a_ were obtained as described in the Experimental section.

c These *k*_nuc_ values were obtained from plots of *k*_obs_ (given as ESI) *versus* nucleophile concentration, as described in the Experimental. Each *k*_obs_ value is an average of duplicate runs with a deviation of ±3%; data for entries 10–12 were obtained by the initial rate method (see text) and are subject to an uncertainty of ±5%.

d Values of *k*_nuc_ in water at 25 °C (*k*^water^_nuc_) were taken from our previous work in ref. 2.

e These (*k*^aqEtOH^_nuc_) are *k*_nuc_ values in 70% water–30% ethanol (this work).

**Fig. 3:**
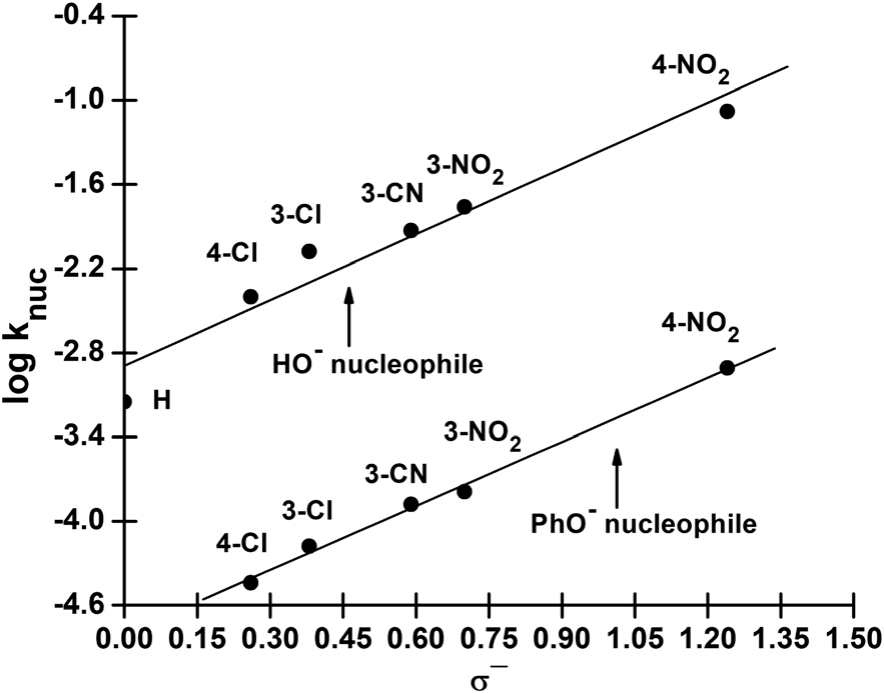
Hammett (log *k*_nuc_*vs. σ*^−^) plot for the reactions of 3a–g with HO^−^ and PhO^−^ in 70% water–30% ethanol (v/v) at 25 °C.

**Fig. 4:**
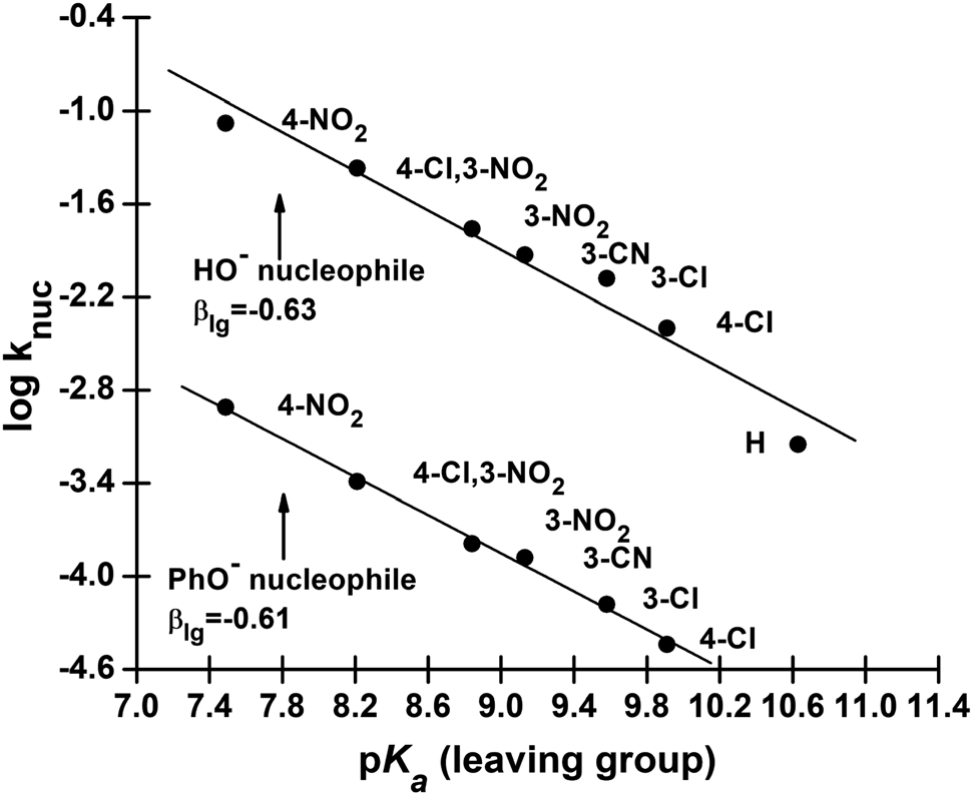
Plots of log *k*_nuc_*vs.* p*K*_a_ (leaving group) for the reactions of HO^−^ (upper plot) and PhO^−^ (lower plot) with aryl dimethylphosphinothioate esters 3a–g in 70% water–30% ethanol (v/v) solvent at 25 °C. The lines for HO^−^ and PhO^−^ are defined by eqn (6) and (7), respectively.

We have also measured polar substituent effects in the reactions of 3a–g with the nucleophiles HO^−^ and PhO^−^ at 25 °C in 70% water–30% ethanol. The pseudo-first-order rate constants, *k*_obs_, calculated from linear plots of log(*A*_∞_ − *A*_*t*_) *versus* time, are also given as ESI.† The resulting *k*_nuc_ values are collected in Table 3. These *k*_nuc_ values were used to construct Hammett plots, using *σ*, *σ*^0^ and *σ*^−^ constants from the literature;^36,37^ the plot with *σ*^−^ constants is displayed in Fig. 3. The different *ρ* values obtained from Hammett plots using *σ*, *σ*^0^ and *σ*^−^ constants, along with their corresponding *R* values, are assembled in Table 4. Brønsted-type plots of log *k*_nuc_*versus* p*K*_a_ (leaving group) for the reactions of 3a–g with HO^−^ and PhO^−^ were also constructed from the data in Table 3 and shown in Fig. 4.

**Table 3:** Second-order rate constants (*k*_nuc_)^a^ for the reactions of HO^−^ and PhO^−^ with a series of substituted aryl dimethylphosphinothioates in 70% water–30% ethanol (v/v) at 25 °C

Entry	Leaving group	p*K*_a_^b^	10^4^*k*_nuc_^c^/M^−1^ s^−1^	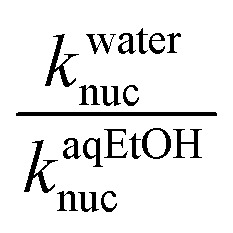 d ^,^ e
**(A) Nucleophile = HO** ^**−**^				
1	4-NO_2_PhO^−^	7.48	830	79.9
2	4-Cl, 3-NO_2_PhO^−^	8.21	427	70.0
3	3-NO_2_PhO^−^	8.84	175	94.9
4	3-CNPhO^−^	8.92	118	105.9
5	3-ClPhO^−^	9.58	83.6	57.4
6	4-ClPhO^−^	10.02	40.1	109.7
7	PhO^−^	10.54	7.10	323.9

**(B) Nucleophile = PhO** ^**−**^				
1	4-NO_2_PhO^−^	7.48	12.3	13.5
2	4-Cl, 3-NO_2_PhO^−^	8.21	4.11	88.8
3	3-NO_2_PhO^−^	8.84	1.61	75.8
4	3-CNPhO^−^	8.92	1.32	83.3
5	3-ClPhO^−^	9.58	0.67	
6	4-ClPhO^−^	10.02	0.37	

a These rate constants were measured at ionic strength, *I* = 1.0 M (KCl).

b Values of p*K*_a_ were obtained as described in the Experimental section.

c These *k*_nuc_ values were obtained from plots of *k*_obs_ (given as ESI) *versus* nucleophile concentration, as described in the Experimental, in which each *k*_obs_ value is the average of duplicate runs with a deviation of ±3%.

d Values of *k*_nuc_ in water at 25 °C (*k*^water^_nuc_) were taken from our previous work.^2^

e These (*k*^aqEtOH^_nuc_) are *k*_nuc_ values in 70% water–30% ethanol (this work).

**Table 4:** Hammett *ρ* values with correlation coefficients (*R*) for the leaving group variation in the reactions of HO^−^ and PhO^−^ with some dimethylphosphinothioates in water at 25 °C (see text)

Substituent constant^a^	*ρ* value (*R*)	
	HO^−^	PhO^−^
*σ*	2.22 ± 0.24 (0.956)	2.25 ± 0.25 (0.890)
*σ* ^0^	2.21 ± 0.21 (0.967)	2.33 ± 0.23 (0.920)
*σ* ^−^	1.57 ± 0.21 (0.968)	1.52 ± 0.15 (0.998)

a Values of these substituent constants were taken from C. Hansch, A. Leo and R. W. Taft, *Chem. Rev.*, 1991, **91**, 165 and R. W. Taft, *J. Phys. Chem.*, 1960, **64**, 1805.

A variety of phosphinate and phosphinothioate esters have been shown^2–4,14,15,17–25,38^ to generally react with anionic oxygen nucleophiles in water, ethanol and water-containing binary solvents *via* nucleophilic attack at the substrate P centre, the only apparent exception to this general trend being the diphenyl phosphinate series of substrates, 1,^17,25^ whose nucleophilic reactions proceed by a general base-catalyzed pathway. For the specific case of the reaction of 3a with phenoxide and 4,4,4,3,3,2,2-heptafluorobutanol in water, the solvent isotope effect values of 1.00 ± 0.02 and 1.01 ± 0.05, respectively, reported^2^ for these nucleophiles are consistent with the behaviour of these bases as nucleophiles rather than as general bases. The fact that hydroxide ion (*vide infra*) and all the alkoxide nucleophiles employed in the present study define the same Brønsted plot as the phenoxides (see Fig. 2 and 3) indicates that the reactions involving these other nucleophiles also occur *via* nucleophilic attack at phosphorus. The reactions of these substrates in 70% water–30% ethanol herein reported are, as a consequence, discussed in terms of nucleophilic attack at the P centre to generate the reaction products.

### Linear free energy relationship (LFER) correlations

#### (i) Hammett correlations

As revealed by Table 4, Hammett plots for the reaction of HO^−^ constructed with *σ*^−^, *σ* and *σ*^0^ constants give similar values of *R* and are therefore not helpful as diagnostic tools for discussing the mechanism of the reaction involving this nucleophile. However, the Brønsted plots considered below show that the reactions of the two nucleophiles, HO^−^ and PhO^−^, follow the same mechanism. On the other hand, the Hammett plot for the reaction of PhO^−^ gives a better correlation with *σ*^−^ constant than with *σ* and *σ*^0^ constants, with *ρ*^−^ = 1.52 ± 0.15 (*R* = 0.998), similar to the value of 1.57 ± 0.21 (*R* = 0.968) obtained for the same correlation when HO^−^ is the nucleophile. This value of *ρ*^−^ is higher than the value of 1.05 ± 0.27 reported^2^ for the reaction of this nucleophile with the same substrate series in water, indicating a higher sensitivity of the reaction to electronic effects in the less polar aqueous ethanol medium. The fact that a better correlation is obtained with *σ*^−^ substituent constants for the reaction of PhO^−^ than with their *σ* or *σ*^0^ counterparts points to a mechanism in which the fission of the P–OAr bond is involved in the rate-limiting step of the reaction. This means that the rate-limiting step of the reaction could be the decomposition to products of the pentacoordinate intermediate formed in an associative pathway (A) or the concerted formation and cleavage of bonds in pathway (B) in Scheme 1. Additional diagnostic criteria would therefore be required to settle for either of these two mechanisms.

#### (ii) Brønsted-type correlations

Brønsted-type correlations are used routinely in mechanistic chemistry to probe the properties of the TS of many types of organic reactions.^12,15,28,39,40^ In particular, *β*_nuc_, *β*_lg_ and other parameters derivable from these exponents are useful in estimating TS structures and in assessing how the TS responds to reaction variables such as change of substituents, solvents and other reaction conditions. An important caveat for the construction of valid Brønsted correlations in nucleophilic reactions is that the nucleophiles utilized in such studies should be structurally related and endowed with the same atom at their nucleophilic sites.^23,28,39,40^

**Scheme 2:**
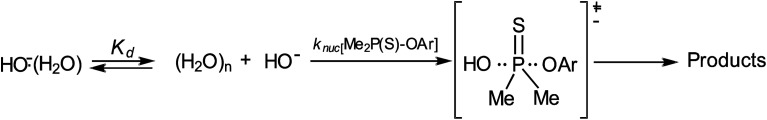


The Brønsted plot in Fig. 2 is linear across the p*K*_a_ range of 5.86–13.53 with phenoxides and alkoxides as nucleophiles; the regression line is defined by eqn (4), from which *β*_nuc_ = 0.44 ± 0.03 (*R* = 0.967) is obtained. The fact that the linear plot in Fig. 1 spans the p*K*_a_ range of 5.86–13.53, which bestrides the p*K*_a_ of the leaving group (4-nitrophenol p*K*_a_ in 70% water–30% ethanol (v/v) = 7.48 (ref. 41)), is good evidence that the reaction of 3a involving these nucleophiles proceeds by a concerted mechanism, *via* a single TS.^23,25^ Unlike the reaction in water, in which the strongly basic nucleophiles, hydroxide ion and alkoxides, follow a different linear correlation with a diminished slope relative to the slope described by the phenoxides,^2^ only the point for HO^−^ (p*K*_a_ = 16.60) shows a negative deviation in the aqueous ethanol solvent. Inclusion of the point for HO^−^ in the plot in Fig. 1 gives a poorer correlation (*R* = 0.953) with *β*_nuc_ = 0.37 ± 0.06. A possible explanation for the behaviour of HO^−^ is that this oxyanion is sufficiently basic to be solvated in the aqueous ethanol solvent, which results in its decreased nucleophilic reactivity, a phenomenon known as solvational imbalance discussed by Jencks^42,43^ and Bernasconi.^44^ The idea that HO^−^ in aqueous ethanol solvent is solvated by a combination of two or three ethanol and water molecules has long existed in the literature.^45^ Such solvational imbalances manifest as negative deviations of log *k*_nuc_ from the straight lines defined by less basic nucleophiles in Brønsted plots, according to eqn (5).^42,43^ The requirement for desolvating the nucleophile before a subsequent nucleophilic attack on the substrate is conveyed by Scheme 2, in which *K*_d_ is the equilibrium constant for the desolvation step. It is also possible for partial desolvation of the nucleophile and nucleophilic attack on the substrate to occur concurrently.^44^ Both models would result in decreased nucleophilicity of strongly basic nucleophiles in hydroxylic solvents, as observed for HO^−^.

4 log *k*_nuc_ = (0.44 ± 0.03)p*K*_a_ − (7.22 ± 0.33)

5 Δlog *k*_nuc_ = (1 − *β*_nuc_)log *K*_d_

Rate data for the dependence of *k*_nuc_ on the basicity of the LG for the reactions of 3a–g with the nucleophiles HO^−^ and PhO^−^ given in Table 3 are plotted in Fig. 3. The straight lines in this figure are defined by eqn (6) and (7) for HO^−^ and PhO^−^ as nucleophiles, respectively, which give *β*_lg_ = −0.63 ± 0.15 (*R* = 0.980) for HO^−^ and −0.61 ± 0.04 (*R* = 0.998) for PhO^−^. Since *β*_lg_ is similar for both nucleophiles, the average value of *β*_lg_ = −0.62 is utilized in the following discussion. The similarity in *β*_lg_ for the reactions of these two nucleophiles, as we argued previously,^2^ is a manifestation of the fact that nucleophilic attack by the weakly to moderately basic PhO^−^ nucleophiles and the strongly basic RO^−^ and HO^−^ nucleophiles at the phosphorus centre reduces the effective charge on the departing aryloxy group in the TS to the same extent for both sets of nucleophiles. Stated alternatively, even though log *k*_nuc_ for HO^−^ deviates from the Brønsted correlation in Fig. 2 due to the stabilization of the ground state for its reaction by solvation, the amount of bonding in the TS is the same for this and other nucleophiles.

6 log *k*_nuc_ = (−0.63 ± 0.15)p*K*_a_ + (3.80 ± 0.26)

7 log *k*_nuc_ = (−0.61 ± 0.04)p*K*a + (1.68 ± 0.23)

The Brønsted-type correlations undertaken above have produced Brønsted parameters *β*_nuc_ = 0.44 and *β*_lg_ = −0.62 for the reactions of 3a–g with anionic oxygen nucleophiles in 70% water–30% ethanol (v/v) solvent. Relative to their values in water (*β*_nuc_ = 0.47, *β*_lg_ = −0.53),^2^ these *β*_nuc_ and *β*_lg_ values point to very slightly reduced bond formation and increased bond breaking in the TS of the reaction in this solvent, *i.e.* a looser TS in aqueous ethanol. The values in aqueous ethanol may also be compared with *β*_nuc_ = 0.41 and *β*_lg_ = −0.47 obtained^26^ for the reactions of oxyanionic nucleophiles with aryl dimethylphosphinothioate esters, 2, the oxygen analogues of 3, in water.

### Effective charge distribution and transition state structure

The methodology of Jencks^28,46^ and Williams^37,47^ is used here to map the effective charge, *ε*_TS_, on the entering nucleophile and LG in the TS, to aid a discussion of the TS structure, using the values of *β*_nuc_ (0.44) and *β*_lg_ (−0.62) evaluated above for the reaction in aqueous ethanol. This exercise entails the estimation of *β*_eq_, on the conditions that (i) the values of *β*_nuc_ and *β*_lg_ are measured for reactions which are the microscopic reverse of each other, and (ii) both parameters are associated with the same rate-limiting step. The reaction under discussion satisfies these two conditions. The parameter *β*_eq_, already defined in eqn (2) and calculated as +1.06, measures the overall charge change on the nucleophile (or the leaving group) between the reactant and product states for the overall reaction.

**Fig. 5:**
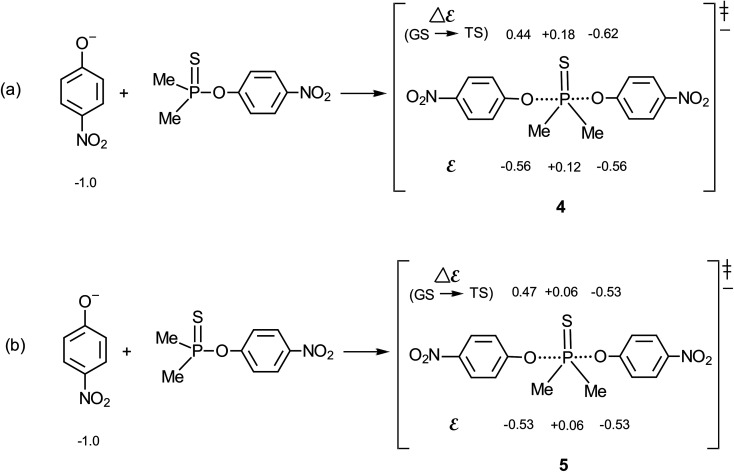
Effective charges in the TS of the identity reaction in which the nucleophile = leaving group = 4-nitrophenoxide: (a) this work, in 70% water–30% ethanol; and (b) in water, see ref. 2.

The effective charges on O of the attacking nucleophile and O of the departing nucleofuge in going from the ground state to the TS, Δ*ε* (GS → TS), are equal to *β*_nuc_ (0.44) and *β*_lg_ (−0.62), respectively, leaving a charge imbalance between the attacking and leaving group O of +0.18. This charge is probably offset by −0.18 charge unit from the thiophosphinoyl group in order to maintain overall charge neutrality. The quantity *β*_nuc_ is related to the effective charge on the nucleophile in the TS according to eqn (8), where *ε*_R_ is the effective charge on the nucleophile in the ground state which, by definition, is −1. For the system under consideration, therefore, *ε*_TS_ = −0.56. The total charge of the nucleophile plus substrate in the ground state is −1, whilst the charges on the entering and departing groups in the TS sum up to −1.12, leaving a charge balance of −0.12, which is probably offset by +0.12 charge unit on the thiophosphinoyl group in order to maintain overall charge neutrality. The effective charges as evaluated above are shown in the TS, shown as structure 4 for the symmetrical identity reaction in Fig. 5a, in which nucleophile = leaving group = 4-nitrophenoxide. This charge distribution shows that the Me_2_P=S moiety carries significant positive charge in a TS that is trigonal bipyramidal, probably with lengthened axial distances to the entering and departing oxygens.^23^ The effective charges for the TS in water,^2^5, are shown in Fig. 5b, for comparison. It is important to note that the Me_2_P=S fragment in the TS carries double the amount of charge than it carries in pure water.

8 *ε*_TS_ = *β*_nuc_ + *ε*_R_

The Leffler indices,^26^ which measure the extent of bond formation, *α*_bf_, and the extent of bond rupture, *α*_br_, in the TS, already defined in eqn (1), are calculated by normalizing the Brønsted parameters *β*_nuc_ and *β*_lg_ with the *β*_eq_ value. This procedure yields the values of 0.42 and −0.58 for *α*_bf_ and *α*_br_, respectively, which show that in 70% water–30% ethanol, bond fission is ahead of bond formation in a TS that is looser in this solvent than in water in which *α*_bf_ = 0.47 and *α*_br_ = −0.53.^2^ The Leffler indices in aqueous ethanol locate the TS in this solvent at *T*^‡^_2_ further down along the tightness diagonal^48^ from *T*^‡^_1_, the TS in water. This outcome is explored further below by considering possible solvent/solvation effects on the reaction in the aqueous ethanol solvent, relative to water.

### Solvent/solvation effects on rates and transition state structure

The Hughes–Ingold theory of solvent action^49^ generally predicts slower bimolecular nucleophilic substitution reactions of aliphatic substrates in polar solvents such as water than when they are conducted in less polar counterparts, if the nucleophile is negatively charged and the substrate is neutral, a charge-type 1 reaction. This is attributed to the stronger solvation of the initial state than the TS in polar solvents, hence rate accelerations occur when the solvent is changed to less polar solvents which destabilize the initial state by desolvating the ions, thus making the nucleophile more reactive. Since charge-type 1 reactions involve charge dispersal in the TS, the effect of a change, from a polar solvent to a less polar one or *vice versa*, is predicted to be relatively modest, as is usually observed experimentally.^50^

The situation with the reaction of neutral phosphorus esters with charged nucleophiles is however different. Organic solvents decrease the rates of bimolecular reactions of phosphorus esters relative to their polar counterparts because the former group of solvents stabilize the initial states of the reactions by decreasing the activity coefficients of the neutral substrates. This inhibitory effect more than offsets the rate-enhancing IS destabilization (desolvation) of the anionic nucleophiles by organic co-solvents, leading to slower reactions.^51^ In some cases, the stabilization of the IS by the less polar solvent is partially offset by the stabilization of the TS.^52^ The hydrolysis of 4-nitrophenyl diphenylphosphate^52^ and a number of phosphinate esters^51,53,54^ have been shown to be faster in aqueous solvents than in binary aqueous organic solvents. The reactions of 3a–g with the different oxyanionic nucleophiles in 70% water–30% ethanol reported in this paper are all slower than the corresponding reactions in water,^2^ the ratio of the second-order rate constants of these reactions in water and aqueous ethanol solvent, 
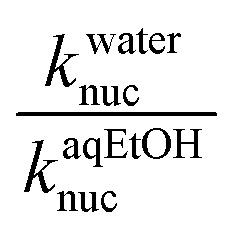
, ranging from 13.5 to 323.9 at 25 °C (see Tables 1 and 2), consistent with the effects of solvents on the reactions of phosphinate esters pointed out above. These rate differences translate to activation energy differences, Δ*E*_a_, in the range of 6.4–14.3 kJ mol^−1^, pointing to the significant effects which solvation factors could confer on these reactions. Water and 70% water–30% ethanol have dielectric constant (*D*) values of 80.2 and 64.0, respectively, at 20 °C,^29,30^ pointing to the difference in the polarities of these two solvents.

**Fig. 6:**
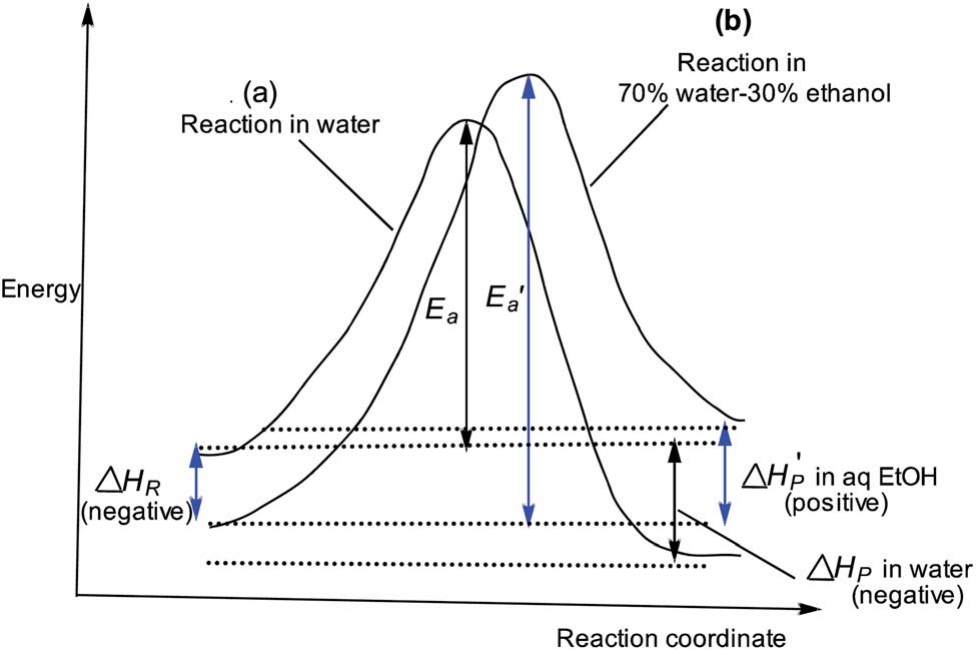
Reaction coordinate diagrams showing qualitatively: (a) reaction coordinate diagram for reaction in water with activation energy *E*_a_ and a negative Δ*H*_P_ (exothermic); (b) reaction coordinate diagram for reaction in 70% water–30% ethanol, showing 
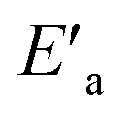
 for the slower, more endothermic reaction (positive 
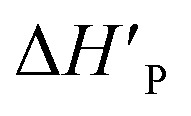
) in which the TS is more product-like than it is in water, according to the Hammond postulate, and the initial state is stabilized (negative Δ*H*_R_) by a decrease in the activity coefficient of the substrate (see text).

The slower reaction of 3a–g with oxyanionic nucleophiles in 70% water–30% ethanol are more endothermic (more positive or less negative 
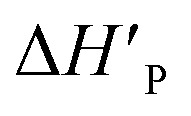
) and, according to the Hammond postulate, should have a TS that is more product-like (*i.e.* later) than the TS in water. The effects of change in solvent polarity on the initial and final states of the reaction may be sketched as shown in the hypothetical reaction coordinate diagram in Fig. 6. The IS is stabilized by the less polar solvent by decreasing the activity coefficient of the neutral substrate, which more than offsets the destabilization of the negatively charged nucleophile,^42^ thereby decreasing the energy of the IS (*i.e.* Δ*H*_R_ is negative). The slower reaction in the less polar solvent means that the more product-like TS benefits less than the IS from the stabilization that the solvent brings, the nett result being that the activation barrier in 70% water–30% ethanol, 
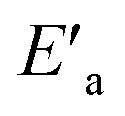
, becomes higher than it is in water, *E*_a_. These considerations show that the change in solvent to one of lower polarity in this instance leads to a more product-like TS for the concerted reaction, which is in qualitative agreement with the direction of the Brønsted exponents calculated above for the reaction in 70% water–30% ethanol.

The change in the TS structure of this reaction in aqueous ethanol, relative to the TS in pure water (*T*^‡^_1_), can also be elucidated by means of the More O'Ferrall–Jencks diagram in Fig. 1. The stabilization of the IS by changing the solvent from water to the less polar 70% water–30% ethanol lowers the bottom left (reactant) corner of Fig. 1, represented by the bold arrow R. The Hammond effect of stabilizing the IS is to raise the upper right (products) corner of Fig. 1 as shown by the bold arrow S. These movements are equivalent to movement of the TS along the concerted reaction coordinate according to the arrow *x* ↔ *y*.^55,56^ The anti-Hammond effect is the movement of the TS in a direction perpendicular to the reaction coordinate as shown by vector *z*, thereby sliding the TS toward looser structures in this solvent than it is in pure water.

The outcome of this study, *i.e.* a looser TS for the reaction of 3 with oxygen anionic nucleophiles in 70% water–30% ethanol (*β*_nuc_ = 0.44, *β*_lg_ = −0.62) than in water (*β*_nuc_ = 0.47, *β*_lg_ = −0.53), is evidently the resultant of two opposing effects, namely: (i) the effect of the increase in the basicity of the nucleophiles and LGs in going from water to 70% water–30% ethanol which results in TS structures that are tighter in this solvent than in water, and (ii) solvent/solvation factors in the less polar solvent which result in a lowering of the energy of the IS of the reaction and, consequently, a slower reaction with a more product-like TS than measured in water. The fact that the nett movement of the TS is in the direction of looser TS shows that the effect due to solvent/solvation factors dominates over the effect of changes in nucleophile and LG basicity in determining TS structure in the less polar solvent. The solvent/solvation effect operational in the nucleophilic reactions of phosphinothioate esters with anionic oxygen nucleophiles in aqueous ethanol reported in this study has a different origin and manifestation from that reported in thiophosphoryl transfer from phosphorothioates to anionic oxygen nucleophiles in water,^57^ or in phosphoryl transfer from substituted phenyl phosphates and phosphorylated pyridine to amines and pyridines in water,^58,59^ or in acyl transfer between sulphur and oxygen nucleophiles in water,^60^*etc.* In these literature examples, the observed solvation effect is due to the stabilization of the nucleophile in the IS by the solvent water which manifests in curved Brønsted correlations due to decreased rates, with little or no effect on the TS structure, whereas in the present study, stabilization of the neutral substrate in the IS in the presence of the organic co-solvent manifests in reduced reaction rates and altered TS structure due to Hammond and anti-Hammond movements of the TS.

The present data are insufficient to enable a determination of the cross-interaction coefficient, *p*_*xy*_ (see eqn (3)), for this reaction, as a prelude to giving a quantitative expression to the effect of change of the basic strength of the nucleophiles and LGs on TS structure, which has been discussed qualitatively above. The value of this parameter is known for the nucleophilic reactions of some phosphorus esters. It has been shown^23^ that *p*_*xy*_ = 0.072 for the identity reaction of 4-nitrophenoxide with 1a in water. Since steric factors present in the reaction of 1a with nucleophiles are absent in 3a, the value of *p*_*xy*_ for the nucleophilic reactions of 1a may not serve as a reasonable model for this parameter in the reaction of the latter substrate. Values of *p*_*xy*_ for phosphoryl transfer from phosphorylated pyridines to a variety of nucleophile types have been measured by Jencks and co-workers, to obtain smaller values of *p*_*xy*_ = 0.013 for transfer to anionic oxygen nucleophiles,^11*c*^*p*_*xy*_ = 0.014 for transfer to pyridines and primary amines,^11*b*,59^ and *p*_*xy*_ ∼ 0.02 for transfer to a variety of amines.^36,59^ The reaction of phosphate esters with pyridine occurs with a value of *p*_*xy*_ = 0.043.^61^ The charge type of these reacting systems are dissimilar to the present system which involves a reaction between a neutral substrate and an anionic nucleophile. Khan and Kirby^62^ obtained the values of *p*_*xy*_ in the range of 0.058–0.069 for the attack of nucleophiles, including oxyanions, on neutral aryl phosphate triesters, while the *p*_*xy*_ values measured for the hydrolysis of epimeric 2-(aryloxy)-2-oxydioxaphosphorinanes in aqueous dioxane by Rowell and Gorenstein^63^ lie in the range of 0.030–0.040. These observations suggest that *p*_*xy*_ for the present system is likely to be modest, probably of the same order of magnitude as measured for the reactions of neutral substrates with anionic nucleophiles cited above. Since Δp*K*_a_ of the nucleophiles and LGs used in this study in going from water to 70% water–30% ethanol is narrow, it is evident that the magnitude of the changes in *β*_nuc_ and *β*_lg_ values, *i.e.* Δ*β*_nuc_ and Δ*β*_lg_, caused by basicity changes in the nucleophiles and LGs is small in magnitude. In other words, the change in TS structure assignable to the effect of Δp*K*_a_ of nucleophiles and LGs, according to (i) above, is expected to be small. On this score, solvent and solvation factors arising from the change in solvent polarity in going from water to aqueous ethanol appear to be largely responsible for the changes in *β*_nuc_ and *β*_lg_ measured in this study, *i.e.* Δ*β*_nuc_ = 0.03 and Δ*β*_lg_ = −0.09. The nett outcome is a looser TS in the less polar solvent. It would be of mechanistic interest to explore how larger changes in nucleophile and LG basicity and further decreases in solvent polarity, both of which are realisable through increases in the ethanol content of the binary aqueous solvent, would further affect the TS structure of this concerted reaction. Such a mapping of the TS^64^ of the reaction, which may provide an insight into the spectrum of the reaction transition states that lie in the continuum of polarities between the extremes of the two solvents, water and ethanol, obtained by a systematic increase in the quantity of one component of the binary mixture at the expense of the other component, is under active consideration.

The significance of the present result, in which lowering the polarity of the reaction medium has given rise to a looser TS for a nucleophilic substitution at a phosphorus centre involving oxyanions, is that it highlights the need for caution in the extrapolation of results from uncatalyzed nucleophilic reactions of phosphorus substrates in water to analogous phosphoryl transfer reactions in biological systems. The microenvironment of the active site in which enzymatic reactions occur is known to be packed with macromolecules and other solutes.^65,66^ It is, by and large, a comparatively less polar medium than water and is known to promote specific interactions of ground states and transition states of reacting entities with endogenous amino acid residues and other solutes in the system.^62,67^ Such media are characteristically different from the dilute aqueous solutions in which the determination of transition states of the nucleophilic reactions of phosphorus ester substrates is mostly executed in analogous chemical reactions and may, therefore, affect mechanisms and TS structures in enzymatic reactions in ways that would not be predicted from analogous chemical reactions.

## Experimental

### Materials

Distilled and deionized water was degassed under vacuum. 1,4-Dioxane was purified by passing it through an alumina column in order to remove any adventitious peroxides. Anhydrous ethanol was first distilled before it was used to make the reaction medium, 70% water–30% ethanol (v/v), used in the kinetics experiments. The parent phenols and alcohols used to generate the nucleophiles were commercial products which were recrystallized before use. Analytical grade sodium hydroxide was standardized with phenolphthalein before use. The buffer materials CAPS, MOPS and Bis–Tris were all of analytical reagent grade. The syntheses of the substrates 3a–g have been described previously.^2^ The compounds were carefully stored in the refrigerator and were recrystallized to obtain melting points which agreed with literature values, before use. Additionally, experimental absorbances of the reaction solutions were measured at the end of each kinetic experiment (see below). In all cases the experimental absorbances at infinity agreed with the theoretical ones; these served as indirect checks on the integrity of the substrates.

### Determination of the p*K*_a_ values of the nucleophiles in 70% water–30% ethanol

The p*K*_a_ values of the hydroxide ion, alcoholates and phenolates utilized as nucleophiles and leaving groups in this study were obtained by the Yasuda–Shedlovsky extrapolation and interpolation procedure.^68–70^ The p*K*_a_ values determined by this theoretical method are in perfect agreement with experimentally measured values (see p*K*_a_ values in parenthesis and footnote *e* in Table 1), thereby reinforcing confidence in the extrapolation/interpolation procedure employed in determining the p*K*_a_ values of the nucleophiles in the aqueous ethanol solvent used this study.

### Kinetic measurements

Kinetic measurements were made on a Yuefeng Model 752 spectrophotometer equipped with a Genlab WBH6/FL thermostatic water bath. Reactions with HO^−^ as nucleophile were carried out in CAPS buffer solutions. For alcoholates and phenolates, except 2,4,5-Cl_3_PhO^−^ and 2,3,5,6-F_4_PhO^−^, self-buffered nucleophile (Nuc^−^) solutions were obtained by partial neutralization of their conjugate acids (NucH) with NaOH, in a way that a NucH : Nuc^−^ ratio of 2 : 1 was obtained. For 2,4,5-Cl_3_PhO^−^ and 2,3,5,6-F_4_PhO^−^, appropriate amounts of the nucleophiles were generated by dissolving the parent phenols in MOPS and Bis–Tris buffer solutions, respectively. The details for preparing the nucleophile solutions from these two phenols have been given previously.^2^ All nucleophile solutions used in the kinetics experiments were maintained at ionic strength, *I* = 1 M (KCl). The procedures for the kinetic runs, including the precautions undertaken to ensure pseudo-first-order conditions, and the adjustments made to the experimental procedure to accord with (i) the reactivity of some of the phenolate nucleophiles and (ii) the charged or neutral state of the phenolic products in the reaction solution have been described earlier.^2^ The graphical procedures for reckoning the pseudo-first-order and second-order rate constants, *k*_obs_ and *k*_nuc_, respectively, have also been described.^2^ Each *k*_obs_ value is an average of duplicate runs with a deviation of ±3%; *k*_obs_ values for reactions involving the nucleophiles 2,5-Cl_2_PhO^−^, 2,4,5-Cl_3_PhO^−^ and 2,3,5,6-F_4_PhO^−^ are subject to an uncertainty of ±5%.

## Conclusions

Hammett *ρ*–*σ*^−^ correlation for the reactions of 3a–g with PhO^−^ in 70% water–30% ethanol solution yields a better linear correlation than *ρ*–*σ* or *ρ*–*σ*^0^ correlations, which shows that the rate-limiting step of the reaction involves LG separation. The Brønsted parameters *β*_nuc_ = 0.44 and *β*_lg_ = −0.62 obtained for the reaction of 4-nitrophenyl dimethylphosphinate with oxyanionic nucleophiles in aqueous ethanol are consistent with a single TS that is looser in the binary solvent than in water. The effects of changes in nucleophile/LG basicity and solvent polarity on the TS, analysed with the aid of the More O'Ferrall–Jencks reaction map, show that increased nucleophile and LG basicities in aqueous ethanol move the TS towards more associative structures. This effect is relatively moderate due to the modest *p*_*xy*_ value for this reacting system and the small Δp*K*_a_ span of the nucleophiles and LGs utilized in the study. On the other hand, decreased solvent polarity stabilizes the IS better than the TS, giving rise to reduced rates and Hammond and anti-Hammond effects which move the TS towards more product-like structures. This latter effect is dominant over the effect of increased nucleophile and LG basicities, to yield a looser TS in the aqueous ethanol solvent. Thus a change in the polarity of the solvent water by the addition of ethanol has altered the potential energy surface of the reaction to yield a looser TS in the less polar solvent.

## Author contributions

Ikenna Onyido conceptualized this research, administered the project as the major supervisor, guided data analysis/interpretation and reviewed earlier drafts to produce the final version of the paper. Collins I. Ubochi supervised the experimental work, assisted in data interpretation and revised the first draft of the manuscript. Georgina I. Kalu performed all the experimental work, was involved in data interpretation, prepared the graphics and wrote the first draft of the paper.

## Conflicts of interest

There are no conflicts of interest to declare.

## Supplementary Material

RA-011-D0RA10759J-s001
