# Supplementary material for: Reactions of aryl dimethylphosphinothioate esters with anionic oxygen nucleophiles: transition state structure in 70% water–30% ethanol
Source: RSC Adv. 2021 Feb 25;11(15):8833–45. doi: 10.1039/d0ra10759j (PMC8695247; doi:10.1039/d0ra10759j)
Supplement: RA-011-D0RA10759J-s001 [file RA-011-D0RA10759J-s001.pdf]

Electronic Supplementary Material (ESI) for RSC Advances.
This journal is © The Royal Society of Chemistry 2021

THIOPHOSPHINOYL TRANSFER FROM ARYL DIMETHYLPHOSPHINOTHIOATE ESTERS TO ANIONIC OXYGEN NUCLEOPHILES: THE EFFECT OF SOLVENT POLARITY ON THE TRANSITION STATE†

Georgina I. Kalu,[a] Cornelius I. Ubochi[a] and Ikenna Onyido[b]*

[a]Department of Chemistry, Imo State University, Owerri, Nigeria
[b]Department of Pure and Industrial Chemistry, Nnamdi Azikiwe University, Awka, Nigeria

Electronic Supplementary Information

Table S1

Pseudo-First-Order Rate Constants (k_{obs}/s^{-1}) for the Reaction of 4-Nitrophenyl Dimethylphosphinates (**3a**)[a] with Oxyanionic Nucleophiles in 70% Water-30% Ethanol (v/v) at 25°C[b]

A.	Nucleophile = HO^-						
	$10^4[HO^-]$ /M	5.0	10.0	20.0	30.0	40.0	50.0
	10^4k_{obs}/s^{-1}	2.60	3.10	3.80	4.74	5.64	6.39
B.	Nucleophile = $CHCl_2CH_2O^-$						
	$10^4[CHCl_2CH_2O^-]$ /M	5.0	10.0	20.0	30.0	40.0	50.0
	10^5k_{obs}/s^{-1}	3.04	6.03	12.5	18.3	24.4	30.0
C.	Nucleophile = $CF_3CH_2O^-$						
	$10^4[CF_3CH_2O^-]$/M	5.0	10.0	20.0	30.0	40.0	50.0
	10^5k_{obs}/s^{-1}	2.05	4.27	8.55	13.1	16.6	21.5
D.	Nucleophile = $CF_3(CF_2)_2CH_2O^-$						
	$10^4[CF_3(CF_2)_2CH_2O^-]$/M	5.0	10.0	20.0	30.0	40.0	50.0
	10^5k_{obs}/s^{-1}	1.46	2.55	5.07	7.63	10.1	12.8
E.	Nucleophile = 4-MeOPhO$^-$						
	10^4[4-MeOPhO$^-$]/M	5.0	10.0	20.0	30.0	40.0	50.0
	10^7k_{obs}/s^{-1}	6.15	15.4	27.7	43.1	55.4	70.8
F.	Nucleophile = PhO$^-$						
	10^4[PhO$^-$]/M	5.0	10.0	20.0	30.0	40.0	50.0
	10^7k_{obs}/s^{-1}	6.15	12.3	24.6	36.9	49.2	61.5
G.	Nucleophile = 4-ClPhO$^-$						
	10^4[4-ClPhO$^-$]/M	5.0	10.0	20.0	30.0	40.0	50.0
	10^7k_{obs}/s^{-1}	5.54	9.22	19.4	30.8	40.0	52.3
H.	Nucleophile = 3-CNPhO$^-$						
	10^4[3-CNPhO$^-$]/M	5.0	10.0	20.0	30.0	40.0	50.0
	10^7k_{obs}/s^{-1}	1.76	1.85	5.54	7.39	9.22	12.3
I.	Nucleophile = 4-CNPhO$^-$						
	10^4[4-CNPhO$^-$]/M	5.0	10.0	20.0	30.0	40.0	50.0
	10^8k_{obs}/s^{-1}	8.79	17.6	44.0	61.5	73.9	92.2
J.	Nucleophile = 2,5-Cl_2PhO$^-$						
	10^4[2,5-Cl_2PhO$^-$]/M	5.0	10.0	20.0	30.0	40.0	50.0
	10^8k_{obs}/s^{-1}	7.68	15.4	30.7	44.0	61.5	70.3
K.	Nucleophile = 2,4,5-Cl_3PhO$^-$						
	10^4[2,4,5-Cl_3PhO$^-$]/M	5.0	10.0	20.0	30.0	40.0	50.0
	10^8k_{obs}/s^{-1}	5.13	7.18	16.7	23.0	30.8	38.4
L.	Nucleophile = 2,3,5,6-F_4PhO$^-$						
	10^4[2,3,5,6-F_4PhO$^-$]/M	5.0	10.0	20.0	30.0	40.0	50.0
	10^8k_{obs}/s^{-1}	3.62	5.13	11.2	16.7	23.0	30.8

[a][Substrate] generally in the range of 3.0-5.0 x 10^{-5}M. [b]Reactions were carried out at ionic strength, I = 1.0M (KCl).

Table S2

Pseudo-First-Order Rate Constants (k_{obs}/s^{-1}) for the Reactions of Substituted Aryl Dimethylphosphinates (**3a-g**)[a] with HO- and PhO- as Nucleophiles in 70% Water-30% Ethanol (v/v) at 25°C[b]

**A.**	**Nucleophile = HO$^-$**						
1.	Substrate = **3a**; leaving group = 4-NO_2PhO$^-$						
	10^4[HO$^-$]/M	5.0	10.0	20.0	30.0	40.0	50.0
	$10^4 k_{obs}$/s^{-1}	2.60	3.10	3.80	4.74	5.64	6.39
2.	Substrate = **3b**; leaving group = 4-Cl,3-NO_2PhO$^-$						
	10^4[HO$^-$]/M	20.0	30.0	40.0	50.0		
	$10^4 k_{obs}$/s^{-1}	1.18	1.74	2.11	2.48		
3.	Substrate = **3c**; leaving group = 3-NO_2PhO						
	10^4[HO$^-$]/M	20.0	30.0	40.0	50.0		
	$10^5 k_{obs}$/s^{-1}	8.89	11.2	13.1	24.1		
4.	Substrate = **3d**; leaving group = 3-CNPhO$^-$						
	10^4[HO$^-$]/M	20.0	30.0	40.0	50.0		
	$10^5 k_{obs}$/s^{-1}	6.84	8.14	9.59	10.3		
5.	Substrate = **3e**; leaving group = 3-ClPhO$^-$						
	10^4[HO$^-$]/M	20.0	30.0	40.0	50.0		
	$10^5 k_{obs}$/s^{-1}	6.21	6.93	7.61	8.77		
6.	Substrate = **3f**; leaving group = 4-ClPhO$^-$						
	10^4[HO$^-$]/M	20.0	30.0	40.0	50.0		
	$10^5 k_{obs}$/s^{-1}	4.95	5.58	5.99	5.65		
7.	Substrate = **3g**; leaving group = PhO$^-$						
	10^4[HO$^-$]/M	20.0	30.0	40.0	50.0		
	$10^5 k_{obs}$/s^{-1}	3.47	3.58	3.63	3.68		
**B.**	**Nucleophile = PhO$^-$**						
1.	Substrate = **3a**; leaving group = 4-NO_2PhO						
	10^4[PhO$^-$]/M	5.0	10.0	20.0	30.0	40.0	50.0
	$10^7 k_{obs}$/s^{-1}	6.15	12.3	24.6	36.9	49.2	61.5
2.	Substrate = **3b**; leaving group = 4-Cl,3-NO_2PhO$^-$						
	10^4[PhO$^-$]/M	20.0	30.0	40.0	50.0		
	$10^7 k_{obs}$/s^{-1}	8.25	12.4	16.5	20.6		
3.	Substrate = **3c**; leaving group = 3-NO_2PhO$^-$						
	10^4[PhO$^-$]/M	20.0	30.0	40.0	50.0		
	$10^7 k_{obs}$/s^{-1}	3.23	4.85	6.47	8.07		
4.	Substrate = **3d**; leaving group = 3-CNPhO$^-$						
	10^4[PhO$^-$]/M	20.0	30.0	40.0	50.0		
	$10^7 k_{obs}$/s^{-1}	2.64	3.96	5.27	6.60		
5.	Substrate = **3e**; leaving group = 3-ClPhO$^-$						
	10^4[PhO$^-$]/M	20.0	30.0	40.0	50.0		
	$10^7 k_{obs}$/s^{-1}	1.34	2.00	2.67	3.34		
6.	Substrate = **3f**; leaving group = 4-ClPhO$^-$						
	10^4[PhO$^-$]/M	20.0	30.0	40.0	50.0		
	$10^8 k_{obs}$/s^{-1}	7.25	7.78	15.6	16.8		

[a][Substrate] generally in the range of 3.0-5.0 x 10^{-5}M. [b]Reactions were carried out at ionic strength, I = 1.0M (KCl).
